# Soluble DNAM-1, as a Predictive Biomarker for Acute Graft-Versus-Host Disease

**DOI:** 10.1371/journal.pone.0154173

**Published:** 2016-06-03

**Authors:** Minoru Kanaya, Kazuko Shibuya, Rei Hirochika, Miyoko Kanemoto, Kazuteru Ohashi, Masafumi Okada, Yukiko Wagatsuma, Yukiko Cho, Hiroshi Kojima, Takanori Teshima, Masahiro Imamura, Hisashi Sakamaki, Akira Shibuya

**Affiliations:** 1 Department of Immunology, Faculty of Medicine, University of Tsukuba, Ibaraki, Japan; 2 Department of Hematology, Hokkaido University Graduate School of Medicine, Sapporo, Japan; 3 Hematology Division, Tokyo Metropolitan Cancer and Infectious Diseases Center Komagome Hospital, Tokyo, Japan; 4 Department of Clinical Trial and Clinical Epidemiology, University of Tsukuba, Ibaraki, Japan; 5 Department of Clinical Oncology, Ibaraki Prefectural Central Hospital, Ibaraki, Japan; 6 Ibaraki Clinical Education and Training Center, University of Tsukuba Hospital, Ibaraki, Japan; 7 Life Science Center of Tsukuba Advanced Research Alliance (TARA), University of Tsukuba, Ibaraki, Japan; 8 Japan Science and Technology Agency, Core Research for Evolutional Science and Technology (CREST), University of Tsukuba, Ibaraki, Japan; University of Kentucky, UNITED STATES

## Abstract

Acute graft-versus-host disease (aGVHD) is a major complication of allogeneic hematopoietic stem cell transplantation (allo-HSCT). Because diagnosis of aGVHD is exclusively based on clinical symptoms and pathological findings, reliable and noninvasive laboratory tests for accurate diagnosis are required. An activating immunoreceptor, DNAM-1 (CD226), is expressed on T cells and natural killer cells and is involved in the development of aGVHD. Here, we identified a soluble form of DNAM-1 (sDNAM-1) in human sera. In retrospective univariate and multivariate analyses of allo-HSCT patients (n = 71) at a single center, cumulative incidences of all grade (grade I–IV) and sgrade II–IV aGVHD in patients with high maximal serum levels of sDNAM-1 (≥30 pM) in the 7 days before allo-HSCT were significantly higher than those in patients with low maximal serum levels of sDNAM-1 (<30 pM) in the same period. However, sDNAM-1 was not associated with other known allo-HSCT complications. Our data suggest that sDNAM-1 is potentially a unique candidate as a predictive biomarker for the development of aGVHD.

## Introduction

Allogeneic hematopoietic stem cell transplantation (allo-HSCT) is an important therapeutic option for a variety of hematologic malignancies. Acute graft-versus-host disease (aGVHD) is the most critical complication of allo-HSCT [1]. Despite significant advances in understanding of the pathophysiology of aGVHD, glucocorticoid therapy is still the gold standard for treatment of aGVHD [2]; however, the overall outlook of steroid-refractory aGVHD is generally poor, in that long-term survival is rare [3]. Diagnosis of aGVHD is exclusively based on clinical symptoms and histological analysis of biopsy specimens of aGVHD target organs. However, since clinical symptoms are not objective in some cases and biopsy is invasive for patients, accurate diagnosis of aGVHD is not always easy. Therefore, noninvasive and reliable laboratory tests for diagnosis of aGVHD is urgently needed.

Acute GVHD biomarkers have been identified and validated as promising tools for diagnosis, assessment, and prediction of response to therapy and prognostic risk [4, 5]. Several biomarkers have been identified by the University of Michigan Blood and Marrow Transplant group by using proteomics approach [5]. This approach identified a four-protein biomarker panel for aGVHD diagnosis [6]. In addition, several groups have identified organ-specific aGVHD biomarkers, such as elafin for skin aGVHD, regenerating islet-derived 3-alpha (Reg3-alpha) for gastrointestinal tract aGVHD, and hepatocyte growth factor (HGF) and cytokeratin fragment 18 (KRT18) for liver aGVHD, by using liquid chromatography–tandem mass spectrometry [7–10]. Recent reports demonstrated soluble suppression of tumorigenicity 2 (ST2) and the plasma microRNA signature as predictive indicators of aGVHD resistance to systemic steroid therapy and survival of allo-HSCT patients with aGVHD [10, 11].

The leukocyte adhesion molecule DNAX accessory molecule-1 (DNAM-1, also known as CD226) is a member of the immunoglobulin superfamily and is constitutively expressed on most CD4^+^ T cells, CD8^+^ T cells, natural killer (NK) cells, and monocytes [12, 13]. The ligands for DNAM-1 are CD155 (also known as poliovirus receptor or necl-5) and CD112 (nectin-2) [14, 15], which are broadly distributed on hematopoietic and non-hematopoietic cells, including epithelial and endothelial cells. Interestingly, expression of CD155 and CD112 is upregulated by the DNA damage response pathway in response to chemotherapy [16]. Interaction between DNAM-1 on CD8^+^ T cells and NK cells and its ligand on target cells augments cell-mediated cytotoxicity [12, 17]. Moreover, DNAM-1 plays a pivotal role in CD4^+^ helper T cell differentiation [18]. Thus, DNAM-1 is involved in a variety of T cell functions. Recently, we and other groups independently showed a critical role for DNAM-1 in the development of aGVHD in mice [19, 20].

Here, we identified a soluble form of DNAM-1 (sDNAM-1) in human serum. We examined the relationship between serum levels of soluble DNAM-1 and the development of aGVHD in allo-HSCT patients. The results indicated that sDNAM-1 is a strong candidate as a predictive biomarker for the development of aGVHD.

## Materials and Methods

### Patients and samples

Serum samples were obtained from 78 patients up to three times a week during the 7 days before and 28 days after allo-HSCT at Tokyo Metropolitan Cancer and Infectious Diseases Center, Komagome Hospital, Japan between March 2009 and November 2011. Written informed consent was obtained from each patient and healthy volunteers in accordance with the Declaration of Helsinki. This study was approved by the ethics committee of the University of Tsukuba, Japan and Tokyo Metropolitan Cancer and Infectious Diseases Center, Komagome Hospital. Patient characteristics are listed in Table 1. The data center of the Tsukuba Critical Path Research and Education Integrated Leading (CREIL) Center handled the data compilation and management with source validation. Blood samples were collected into Venoject-II, VP-AS109K blood collection tubes (Terumo, Tokyo, Japan), and then serum samples were isolated and preserved in a deep freezer at -80°C until use.

**Table 1:** Patient characteristics and univariate analysis for acute GVHD.

	Total (n = 71)	Soluble DNAM-1 ≥30 pM (days -7 to 0)	P-Value	Univariate analysis for grade II–IV aGVHD	P-Value
Age					
< 50	36 (51%)	15 (42%)		Ref	-
≥ 50	35 (49%)	9 (26%)	0.155	0.730 (0.279–1.912)	0.522
Sex					
Male	45 (64%)	17 (38%)		Ref	-
Female	26 (36%)	7 (27%)	0.352	2.212 (0.820–5.988)	0.114
Diagnosis					
AML	36 (51%)	9 (25%)		Ref	-
ALL	13 (18%)	4 (31%)		3.636 (0.968–13.700)	0.049
MDS	10 (14%)	4 (40%)	0.195	0.974 (0.212–4.485)	1.000
Others	12 (17%)	7 (58%)		1.623 (0.421–6.256)	0.500
Disease status					
Standard risk	43 (61%)	11 (26%)		Ref	-
High risk	28 (39%)	13 (46%)	0.07	0.383 (0.135–1.089)	0.068
Conditioning regimen					
Myeloablative	51 (73%)	19 (37%)		Ref	-
Reduced intensity	20 (37%)	5 (40%)	0.326	0.199 (0.052–0.762)	0.012
Stem cell source					
Bone marrow	55 (79%)	17 (31%)		Ref	-
PBSC	9 (11%)	4 (45%)		0.171 (0.020–1.489)	0.125
Cord blood	7 (10%)	3 (43%)	0.632	0.200 (0.023–1.774)	0.222
Donor					
Related	12 (17%)	6 (50%)		Ref	-
Unrelated	59 (83%)	18 (31%)	0.315	1.278 (0.345–4.732)	0.713
HLA					
Matched	49 (69%)	14 (29%)		Ref	-
Mismatched	22 (31%)	10 (45%)	0.164	0.839 (0.300–2.348)	0.738
GVHD prophylaxis					
CsA based	20 (28%)	8 (40%)		Ref	-
FK506 based	51 (72%)	16 (31%)	0.489	2.276 (0.718–7.216)	0.157
Soluble DNAM-1 (days -7 to 0)					
<30pM	24 (34%)			Ref	-
≥30pM	47 (66%)			3.662 (1.303–10.287)	0.012

AML indicates Acute myeloid leukemia; ALL, acute lymphoblastic leukemia; MDS, myelodysplastic syndrome; PBSC, Peripheral blood stem cell; CsA, Cyclosporin A; and Ref, reference

### Transplantation procedures

The myeloablative conditioning regimen included a combination of cyclophosphamide with or without cytarabine and either total body irradiation (12 Gy) or busulfan. Fludarabine-based reduced-intensity conditioning regimens, such as fludarabine with or without low-dose total body irradiation (2–4 Gy) combined with busulfan or melphalan, were used in elderly patients (≥55 y.o.) Anti-thymoglobulin was not used in the conditioning regimen. Acute GVHD prophylaxis consisted of the continuous infusion of cyclosporin A or tacrolimus combined with short-term methotrexate, which was administered at a dose of 10 mg/m^2^ on day 1, and 7 mg/m^2^ on days 3, 6, and 11. Acute GVHD was graded as previously described [21]. Prophylaxis against bacterial, fungal, and *Pneumocystis jiroveci* infections consisted of fluoroquinolones (either fluconazole or itraconazole) sulfamethoxazole, and trimethoprim. Cytomegalovirus was monitored weekly by a cytomegalovirus antigenemia assay; patients with positive antigenemia, defined as ≥1 cell/ 50 000 cells, were treated with ganciclovir.

### Definition of disease status and transplant-related complications

Acute leukemia in first or second remission, chronic myeloid leukemia in first or second chronic phase, and myelodysplastic syndrome or myeloproliferative neoplasm without leukemic transformation were defined as standard-risk diseases, and others were defined as high-risk diseases [22]. These definitions are based on previous Japanese study [23]. Diagnosis of documented infections was based on clinical or radiological findings or both [24].

### Antibodies and reagents

Anti-human DNAM-1 monoclonal antibody (mAb) TX25 (mouse IgG1) was generated in our laboratory, and mouse anti-human DNAM-1 polyclonal antibody was purchased from R & D Systems (Minneapolis, MN). In this manuscript, anti-DNAM-1 mAb refers to TX25 unless otherwise stated. Anti-CD3, anti-CD4, anti-CD8, anti-CD14, anti-CD25, anti-CD28 and control IgG1 antibodies and recombinant human interleukin (IL)-2 were purchased from BD Pharmingen (San Diego, CA). Phorbol 12-myristate 13-acetate and ionomysin were purchased from Sigma-Aldrich (St. Louis, MO). Matrix metalloproteinase (MMP) inhibitors, GM6001 and tumor necrosis factor-alpha protease inhibitor (TAPI)-1, were purchased from Merck-Calbiochem (Darmstadt, Germany).

### Enzyme-linked immunosorbent assay

Soluble DNAM-1 in sera was measured by sandwich enzyme-linked immunosorbent assay (ELISA). In brief, 96-well plates were coated with anti DNAM-1 mAb (8 μg/mL, 100 μL/well) for 2 h at room temperature, blocked with blocking buffer (10% FBS in PBS, 100 μL/well) for 2 h at room temperature, and then washed three times with washing buffer (0.05% Tween 20). Human DNAM-1-Fc fusion protein (as a standard) and serum samples were plated at 100 μL/well and then incubated overnight at 4°C. After washing, biotinylated rabbit anti-human DNAM-1 polyclonal antibody was reacted (0.6 μg/mL, 100 μL/well) for 1 h at room temperature. The plates were then washed and incubated with ExtraAvidin peroxidase (Sigma-Aldrich) (1:250 in washing buffer, 100 μL/well) for 30 min at room temperature. Following another washing step, the plates were reacted with OPD (o-phenylene diamine dihydrochloride) tablet (Sigma-Aldrich) for 20 min at room temperature. We stopped the reactions with 12.5 μL of 6N HCl and measured absorbance at 490 nm by using a Spectra Max Microplate reader (Molecular Devices, Sunnyvale, CA). All values were determined in triplicate. All the samples during observation period in each patient were analyzed in one setting at least twice.

### Western blotting

Peripheral blood mononuclear cells (PBMCs) were stimulated by anti-CD3 mAb and anti-DNAM-1 mAb, and then the lysates were immunoprecipitated with anti-DNAM-1 mAb. The immunoprecipitates were immunoblotted with anti-DNAM-1 mAb, as described previously [12].

### Statistical analysis

Groups of experimental data were compared by using the two-tailed Mann–Whitney U-test, and groups of categorical clinical data were compared by using the chi-square test of independence. The log-rank test was used to assess differences in cumulative incidence of aGVHD among patient groups, and a Cox proportional hazards model was used to assess the impact of multiple variables on cumulative incidence of aGVHD. For all tests, *P* values of less than 0.05 were considered statistically significant. All statistical analyses were performed with IBM SPSS Statistics version 21 (SPSS/ IBM, Armonk, NY) and GraphPad Prism 5 (GraphPad Software, San Diego, CA). A biostatistics analyst supported the choice of statistical methods and the statistical interpretation of the data.

## Results

### Identification of soluble DNAM-1

**Fig 1:**
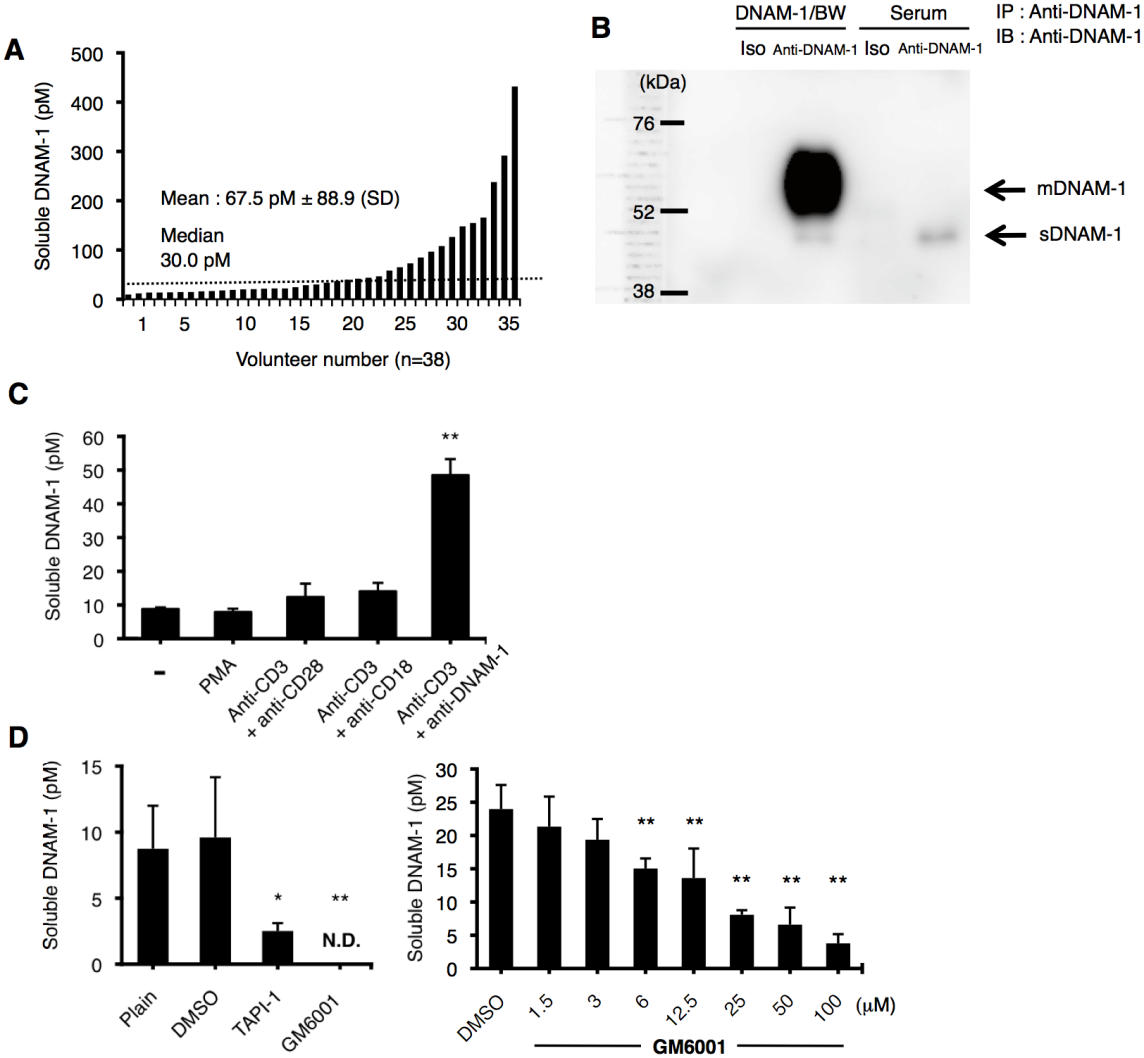
Characteristics of the soluble form of DNAM-1. (A) The soluble form of DNAM-1 (sDNAM-1) in sera from healthy volunteers (n = 38) was measured by sandwich ELISA. (B) sDNAM-1 in human serum was detected by immunoprecipitation (IP) followed by immunoblotting (IB) with mouse anti-human DNAM-1 mAb (TX25). Cell lysate of DNAM-1/ BW transfectant was used as control of membrane DNAM-1 (mDNAM-1). (C) Peripheral blood mononuclear cells (PBMCs) from healthy volunteers were stimulated with the indicated mAbs in the presence of IL-2. sDNAM-1 in the culture supernatant was measured by ELISA on day 7 after the start of culture. (D) PBMCs from healthy volunteers were stimulated with anti-CD3 and anti-DNAM-1 mAbs. On day 3 after the start of culture, the medium was replaced with fresh medium (plain) and a broad-spectrum matrix metalloproteinase (MMP) inhibitor, GM6001 (100 μM) or TAPI-1 (50 μM), was added into the culture. On day 6, sDNAM-1 in the culture supernatant was measured by ELISA. Error bars in all panels represent the mean and standard deviation (SD). * and **, *P* < 0.05 and *P* < 0.01, respectively, compared with the sDNAM-1 concentration in the culture supernatant in the presence of IL-2 alone (C) or DMSO (0.1%) alone (D).

To examine whether a soluble form of DNAM-1 (sDNAM-1) exists in human sera, we established a sandwich ELISA system and evaluated concentrations of sDNAM-1 in 38 healthy volunteers. The mean concentration of sDNAM-1 was 67.5 pM ± 88.9 (SD) and the median was 30.0 pM (Fig 1A). Biochemical analysis of sera by immunoprecipitation and immunoblotting with anti-DNAM-1 mAb (TX25) revealed that the molecular weight of sDNAM-1 was ~45 kDa (Fig 1B), which corresponds to the molecular weight of the extracellular domain of DNAM-1, suggesting that sDNAM-1 might be generated by proteolytic shedding. Furthermore, we never detected shorter than full-length *CD226* mRNA species by RT-PCR analysis, indicating that it is unlikely that sDNAM-1 is encoded by an alternatively spliced isoform of *CD226* mRNA.

To examine how sDNAM-1 was generated, PBMCs obtained from healthy volunteers were stimulated with IL-2 together with either phorbol 12-myristate 13-acetate, anti-CD3 mAb plus anti-CD28 mAb, or anti-CD3 mAb plus anti-CD18 mAb, and the culture supernatants were analyzed for sDNAM-1 by ELISA. Although none of these stimuli significantly increased sDNAM-1 in the culture supernatant compared with the negative control (IL-2 alone), we found that stimulation with IL-2 together with anti-CD3 mAb plus anti-DNAM-1 mAb significantly increased sDNAM-1 (Fig 1C). To address whether sDNAM-1 was generated by proteolytic shedding, we added MMP inhibitors, GM6001 or TAPI-1, into the cultures and found that both MMP inhibitors significantly suppressed the production of sDNAM-1 (Fig 1D). These MMP inhibitors did not affect cell proliferation or activation of T cells after stimulation with anti-CD3 mAb plus anti-DNAM-1 mAb **(**data not shown), suggesting that the MMP inhibitors specifically inhibited MMP activity. Moreover, in RT-PCR analysis, we did not detect any alternatively spliced isoforms of mRNA encoding sDNAM-1 (data not shown). Together, these results suggest that sDNAM-1 is likely generated by proteolytic shedding of membrane-bound DNAM-1 on T cells.

### Association between sDNAM-1 levels and development of aGVHD

**Fig 2:**
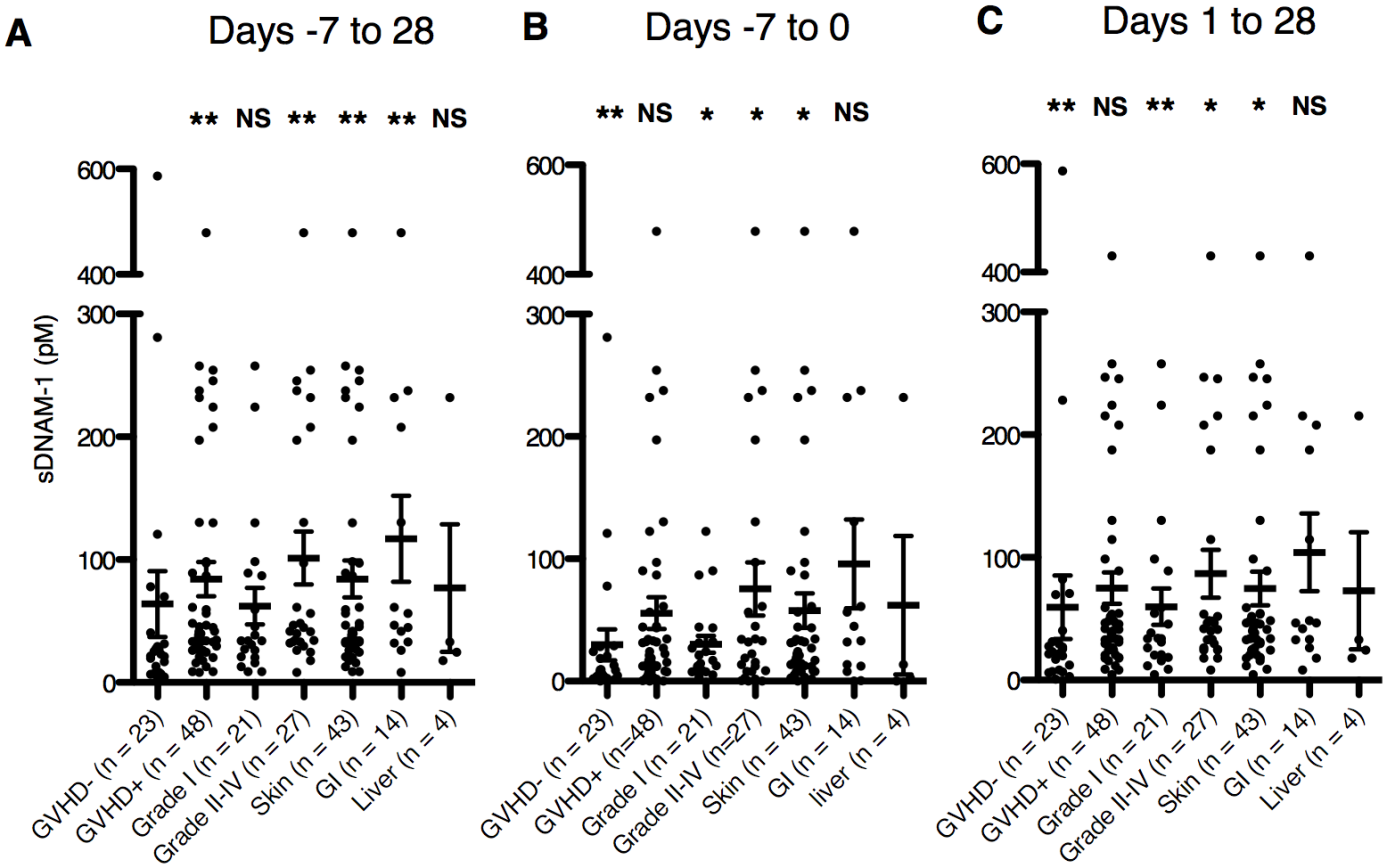
sDNAM-1 levels in patients with or without aGVHD. For the indicated period relative to allo-HSCT (day 0), the maximal concentration of sDNAM-1 was determined separately for patients with or without aGVHD, patients with mild grade (grade I) or severe (grade II–IV) aGVHD, or patients with organ-targeted aGVHD (skin, gastrointestinal [GI], or liver). Error bars in all panels represent the mean and SD. * and **, *P* < 0.05 and *P* < 0.01, respectively, compared with maximal sDNAM-1 levels in patients without aGVHD. NS, not significant.

Because the interaction between DNAM-1 and its ligand plays an important role in the development of aGVHD in mouse models [20, 21], we hypothesized that sDNAM-1 levels increase during development of aGVHD. To examine this idea, we obtained peripheral blood samples from 79 patients undergoing allo-HSCT for the treatment of hematological malignancies up to three times a week for five consecutive weeks around the day of transplantation (days -7 to 28, where day 0 is the day of allo-HSCT) to measure serum sDNAM-1 levels. Eight patients with graft failure were excluded from the analyses due to inadequate observation period. The patients’ profiles are shown in Table 1. We determined the maximal values of sDNAM-1 (max-sDNAM-1) during day -7 to day 28, day -7 to day 0, and day 1 to day 28. The max-sDNAM-1 in each period was significantly higher in aGVHD patients (n = 48) than in non-aGVHD patients (n = 23) (Fig 2). The max-sDNAM-1 of 27 patients with aGVHD at grade II–IV (grade II; n = 22, grade III; n = 4, grade IV; n = 1), but not those with aGVHD grade I (n = 21), was significantly higher than that of non-GVHD patients in each of the observation periods (Fig 2). We also found that the max-sDNAM-1 values in patients with skin aGVHD (n = 43) and gastrointestinal aGVHD (n = 14), but not those with liver aGVHD (n = 4), were significantly higher than that in non-aGVHD patients (Fig 2). These results suggest that the max-sDNAM-1 in the serum is associated with the development of aGVHD.

**Fig 3:**
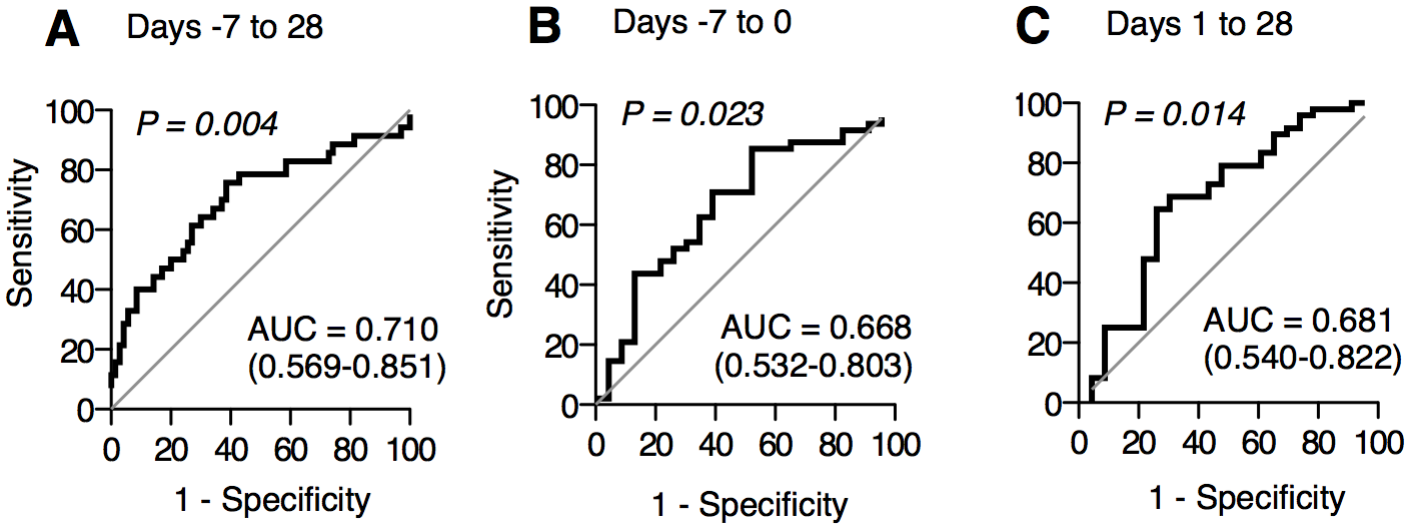
ROC curves for patients subjected to allo-HSCT. ROC curves comparing maximal sDNAM-1 of patients with aGVHD (n = 48) with that of patients without aGVHD (n = 23) for the specified period relative to allo-HSCT (day 0).

We then analyzed the diagnostic ability of the max-sDNAM-1 during day -7 to day 28, day -7 to day 0, and day 1 to day 28 for aGVHD by conducting receiver operator characteristic (ROC) analyses. ROC curves for max-sDNAM-1 in these periods distinguished aGVHD from non-aGVHD with AUC (area under the curve) values of 0.710, 0.668 and 0.681, respectively (Fig 3). These results suggest that the max-sDNAM-1 can be a unique diagnostic biomarker for aGVHD.

**Fig 4:**
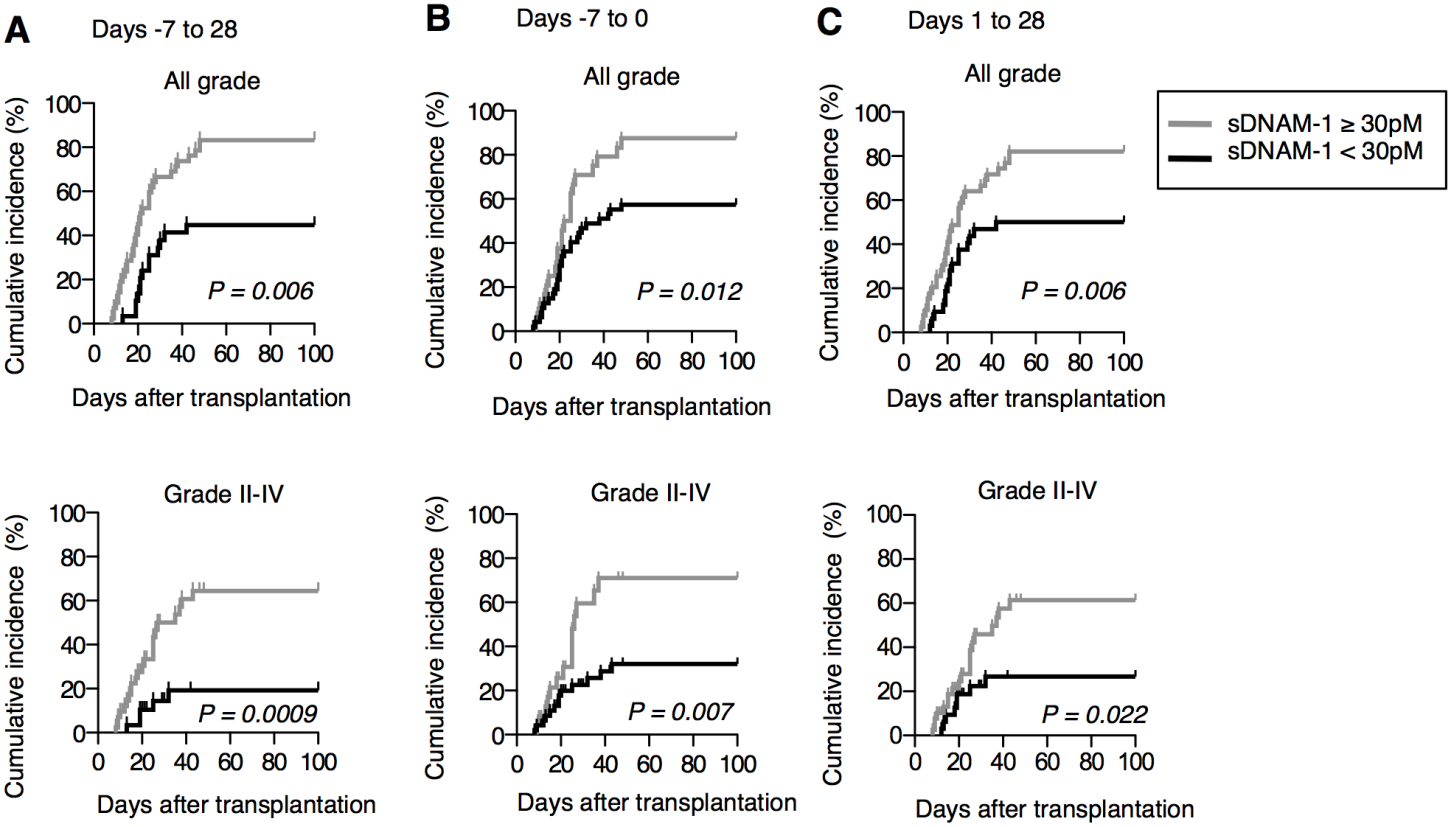
sDNAM-1 levels as a predictor of severe grade aGVHD in patients subjected to allo-HSCT. Patients were divided into two groups according to the maximal concentration of sDNAM-1 (≥30 pM versus <30 pM) in the indicated time periods (A–C) relative to allo-HSCT (day 0). Cumulative incidences of all grade aGVHD and grade II–IV aGVHD were significantly higher in the high sDNAM-1 group than in low sDNAM-1 group in each time period. Log-rank test was performed to assess differences in the cumulative incidence curves.

When we used the criterion of max-sDNAM-1 of 30 pM (median in healthy volunteers) or greater in the period day -7 to day 28 period as a predictor of the development of aGVHD, the sensitivity and specificity were 69.5% and 75.0%, respectively (Table 2**)**. Similar results were obtained for the same criterion for day -7 to day 0 and day 1 to day 28 periods (Table 2**)**. When the data was stratified, max-sDNAM-1 of 30 pM or greater was significantly associated with the development of aGVHD at grade II–IV, but not grade I, and it was significantly associated with the development of skin GVHD and gastrointestinal GVHD, but not liver GVHD (Table 3). The positive predictive value of max-sDNAM-1 of 30 pM or greater for aGVHD at grade II–IV was 0.58 and the negative predictive value of max-sDNAM-1 of 30 pM or less for aGVHD at grade I and non-aGVHD was 0.72 (Table 4). The log-rank test showed that cumulative incidences of both all grades and grade II–IV aGVHD were significantly higher in patients with max-sDNAM-1 of 30 pM or greater when compared to the other patients in any observation period (Fig 4).

**Table 2:** Sensitivity and specificity of soluble DNAM-1.

Cut off: 30 pM	Sensitivity (acute GVHD)	Specificity (acute GVHD)
Days -7 to 28	69.50%	75.00%
Days -7 to 0	43.80%	82.60%
Days 1 to 28	68.80%	69.60%

**Table 3:** Acute GVHD characteristics (univariate analysis).

	Total (n = 71)	Soluble DNAM-1 (≥30 pM (%)) days -7 to 0	Odds ratio	P-Value
No GVHD	23 (33%)	3 (13%)	Ref	-
All acute GVHD	48 (67%)	21 (44%)	5.181 (1.357–20.000)	0.010
Grade I acute GVHD	21 (30%)	7 (34%)	0.971 (0.330–2.857)	0.957
Grade II–IV acute GVHD	27 (38%)	14 (52%)	3.662 (1.303–10.287)	0.012
Skin GVHD	43 (61%)	19 (44%)	3.636 (1.166–11.363)	0.022
Gastrointestinal GVHD	14 (20%)	9(64%)	5.051 (1.456–17.544)	0.007
Liver GVHD	4 (6%)	2 (50%)	1.568 (0.063–6.500)	1.000

Ref indicates reference

**Table 4:** Multivariate analysis for acute GVHD.

Variable	Category	Hazard ratio (All GVHD)	All GVHD	Hazard ratio (Grade II–IV)	P-value (Grade II–IV)
Soluble DNAM-1 (days -7 to 0)	< 30 pM	Ref	-	Ref	-
	≥ 30 pM	2.475 (1.346–4.567)	0.004	2.976 (1.348–6.579)	0.007
Age	< 50	Ref	-	-	-
	≥ 50	0.457 (0.252–0.830)	0.010	-	0.461
Sex	Male	Ref	-	Ref	-
	Female	3.336 (1.826–6.094)	0.000	3.146 (1.417–6.983)	0.027
Disease status	Standard	-	-	-	-
	High	-	0.631	-	0.188
Conditioning	MAC	-	-	Ref	-
	RIC	-	0.971	0.303(0.090–1.021)	0.054

MAC indicates myeloablative conditioning; RIC, Reduced intensity conditioning; and Ref, reference

### Soluble DNAM-1 as a predictive aGVHD biomarker

In a multivariate Cox proportional hazards analysis, age (≥50 years), sex (female), and max-sDNAM-1 (≥30 pM) in the period before allo-HSCT (day -7 to day 0) were significantly positively correlated with all grade aGVHD (Table 4). Furthermore, sex (female) and max-sDNAM-1 (≥30 pM; day -7 to day 0) were also significantly positively correlated with grade II–IV aGVHD (Table 4) in a multivariate analysis.

To investigate the specificity of max-sDNAM-1 as a predictor of aGVHD, we performed univariate analyses for other allo-HSCT complications such as sinusoidal obstruction syndrome, thrombotic microangiopathy, and various infections. In these analyses, max-sDNAM-1 (≥30 pM; day -7 to day 0) was not significantly correlated with the other allogeneic stem cell transplantation complications (S1 Table).

## Discussion

Previous reports showed that serum biomarkers, such as HGF, Reg3-alpha, IL-6, T cell immunoglobulin and mucin domain 3 (TIM3), soluble tumor necrosis factor receptor 1 (sTNFR1), ST2, are useful for diagnosis of aGVHD and prediction of prognosis of aGVHD after therapy including high-dose systemic glucocorticoid treatment [5, 25]. In addition, danger-associate molecular patterns, such as ATP [26] and uric acid [27], produced after conditioning therapy exacerbate aGVHD. These biomarkers were evaluated at the onset of aGVHD. In contrast, here we demonstrated that concentration of serum sDNAM-1 before allo-HSCT was associated with the development of aGVHD, suggesting that sDNAM-1 is potentially a unique biomarker for prediction of the development of aGVHD. However, since the AUC value (0.668) determined by ROC curves for max-sDNAM-1 before allo-HSCT and the PPV had only a mild impact, sole max-sDNAM-1 before allo-HSCT may not be enough for accurate prediction of aGVHD development. Combination of another predictive biomarker, if any, with max-sDNAM-1 before allo-HSCT would help us to treat allo-HSCT patients with additional preemptive therapy for prevention of aGVHD.

Although our results suggest that sDNAM-1 is produced by MMP-mediated proteolytic shedding, the exact mechanism of sDNAM-1 generation remains unclear. In addition, it is also unclear why healthy people showed a broad range of serum sDNAM-1 levels; whether this range in levels is caused by variability in MMP activities among individuals is unknown. Nonetheless, we found that high max-sDNAM-1 in patients was significantly associated with the development with aGVHD. Previous reports demonstrated that MMP activity is critical in the development of aGVHD [28]. Therefore, one of our hypotheses is that recipients with high MMP activity, who might show high serum levels of sDNAM-1, have a high risk of developing aGVHD after allo-HSCT. Alternatively, it may also be possible that sDNAM-1 binds to CD155 on antigen presenting cells or tissues of target organs. Since DNAM-1 shares the ligand CD155 with T cell immunoreceptor with immunoglobulin and ITIM domains (TIGIT) and CD96 [29], it might interfere interaction of CD155 with TIGIT and CD96. In contrast to DNAM-1, TIGIT and CD96 mediate inhibitory signals in T cells and suppress the activation of effector T cells. Therefore, binding of sDNAM-1 to CD155 would cancel the inhibitory signals mediated by TIGIT and CD96 in T cells, resulting in augmentation of T cell activation and exacerbation of aGVHD. In fact, CD155-deficient mice that had received allogeneic hematopoietic transplantation showed increased development of aGVHD [30]. Future studies are required to fully understand the mechanism of generation of sDNAM-1 and its relationship with the development of aGVHD.

The current study has several limitations. First, this study was a single-center retrospective observational study. In this study, female recipient was a risk factor for the development of aGVHD in multivariate analysis, which is contrary to the general consensus. Therefore, the possibility that our study might contain some bias cannot be excluded. To evaluate the reproducibility of the current study, a multicenter, prospective study is required. Second, the physiological function of sDNAM-1 is not well understood. DNAM-1 interaction with the ligands CD155 or CD112 or both plays an important role in a wide array of immune responses in various diseases, including aGVHD [19, 20], tumor immunity [31], infections [13], and autoimmune diseases [32]. sDNAM-1 might affect the interaction between DNAM-1 and these ligands, resulting in modulation of DNAM-1-mediated immune responses.

In conclusion, we identified sDNAM-1 in the sera of healthy people and allo-HSCT patients. Our study showed that sDNAM-1 can be potentially a useful biomarker for prediction of the development of aGVHD. Further investigation is required to elucidate the physiological and pathological functions of sDNAM-1. In addition, a larger prospective study is required to generalize the significance of sDNAM-1 as a aGVHD biomarker. Nevertheless, our concept provides an important framework for developing a method for prediction and evaluation of aGVHD.

## Supporting Information

S1 Table Univariate analysis for other allo-HSCT complications. (DOCX)
