# Supplementary material for: Soluble DNAM-1, as a Predictive Biomarker for Acute Graft-Versus-Host Disease
Source: PLoS One. 2016 Jun 3;11(6):e0154173. doi: 10.1371/journal.pone.0154173 (PMC4892670; doi:10.1371/journal.pone.0154173)
Supplement: S1 Table — (DOCX) [file pone.0154173.s001.docx]

CMV indicates cytomegalovirus.

**Supplementary Table 1. Univariate analysis for other allo-HSCT complications.**
